# Operational data set of a 2 MW natural gas-fired generation engine at shutdown times

**DOI:** 10.1016/j.dib.2020.105369

**Published:** 2020-03-14

**Authors:** Guillermo Valencia Ochoa, Jhan Piero Rojas, Jorge Duarte Forero

**Affiliations:** a Programa de Ingeniería Mecánica, Universidad del Atlántico, Carrera 30 Número 8-49, Puerto Colombia, Barranquilla, 080007, Colombia; b Facultad de Ingeniería, Universidad Francisco de Paula Santander, Avenida Gran Colombia No. 12E-96, Cúcuta, 540003, Colombia

**Keywords:** Gas engine, Electric generator, Stop, Operational data

## Abstract

In this paper, operational data of a natural gas-fired generation engine at 2 MW of power is presented. This engine is used as part of the power supply system of a flexible packaging transformation and conversion plant. This plant, besides having the power supply generated by the engine, receives electrical energy from the network. The data collected from this engine corresponds to measurements taken before, during and after engine stops, whether due to engine maintenance stops, engine failures or external power grid failures. The measurement was made every 10 seconds, and for the storage of these data a data acquisition software was used, which, besides allowing to take these data, shows in real time the electrical behavior of the electrical supply system, as well as the mechanical behavior of the engine.

**Table:** Specifications Table

Subject área	Energy generation
More specific subject área	Internal combustion engines, electric generators
Type of data	Raw, Graphs, figure, table
How data was acquired	Charge pressure sensor Ref. PI E.08.001, boost pressure sensor Ref. E.08-PI-002, mixture temperature sensor Ref. E.08-TI-001, exhaust gas temperature sensor Ref. E.02.001, throttle valve position sensor Ref. E.08-YCI-001
Data format	Raw data and analyzed
Parameters for data collection	The data was obtained before, during and after engine stops due to external grid failure, engine failure or engine maintenance stops.
Description of data collection	The data was collected through the sensors in the engine instrumentation, this instrumentation acquires data every 10 seconds and is presented in real time in the interface of the software of the data acquisition system.
Data source location	Barranquilla, Colombia
Data accessibility	Data is with this article
Related research article	G. V. Ochoa, C. Isaza-Roldan, and J. D. Forero, “A phenomenological base semi-physical thermodynamic model for the cylinder and exhaust manifold of a natural gas 2-megawatt four-stroke internal combustion engine,” Heliyon, vol. 5, no. 10, p. e02700, Oct. 2019.

**Table:** 

**Value of the Data** • The data presented in this paper can be used to evaluate the proper functioning of a natural gas engine, before and after a shutdown. • This data can be used by researchers to characterize the main components of this natural gas engine, and to identify the tendencies of the mean variable under the existence of engine failure, and external power failure. • The raw data presented in this document can be used for modeling natural gas Jenbacher Type 6 engines, and predicting their behavior.

## 1 Data

This article presents operational data of a 2 MW gas-fired generation engine. This engine is part of the electrical energy supply system used in a flexible packaging transformation and conversion company. The 2 MW Jenbacher JMS 612 GS-N.L studied in this paper is the natural gas engine widely used for energy generation reasons worldwide in the industrial sector [1], which is consequence of its adaptability in different industrial applications, such as the oil company, textile, cement, pharmaceuticals, plastics, and paper industries [2].

The energy supply of this company is achieved by taking electrical energy from the network and electrical energy generated by the gas engine.

**Fig. 1:**
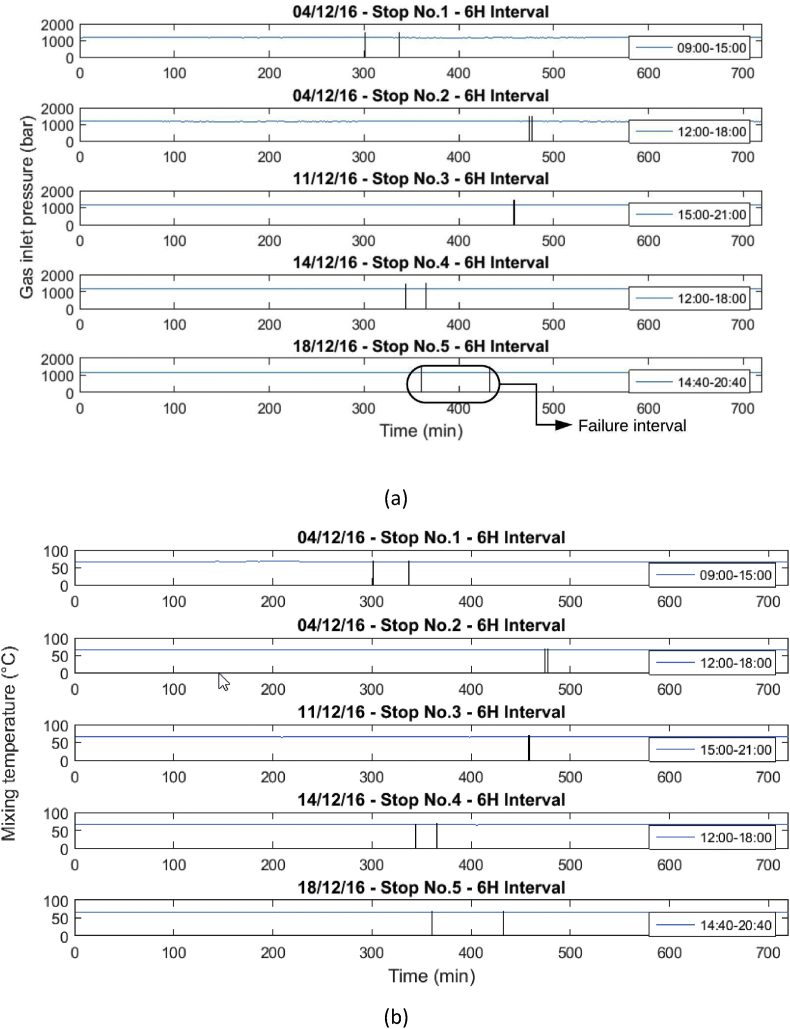
Behavior of the mean engine variables a) gas inlet pressure, and b) temperature of mixture.

**Fig. 2:**
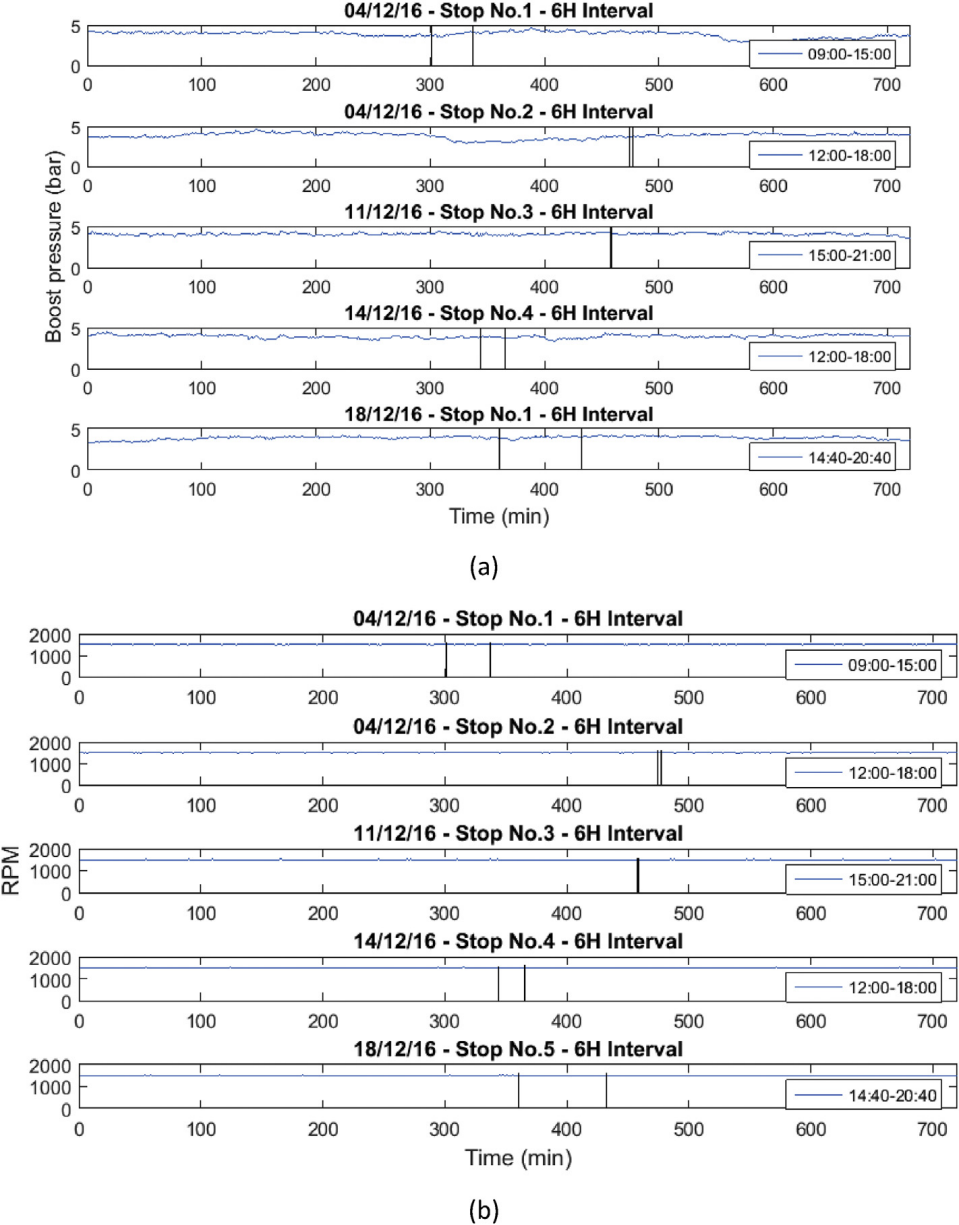
Behavior of the mean engine variables a) Boost pressure, and b) RPM.

**Fig. 3:**
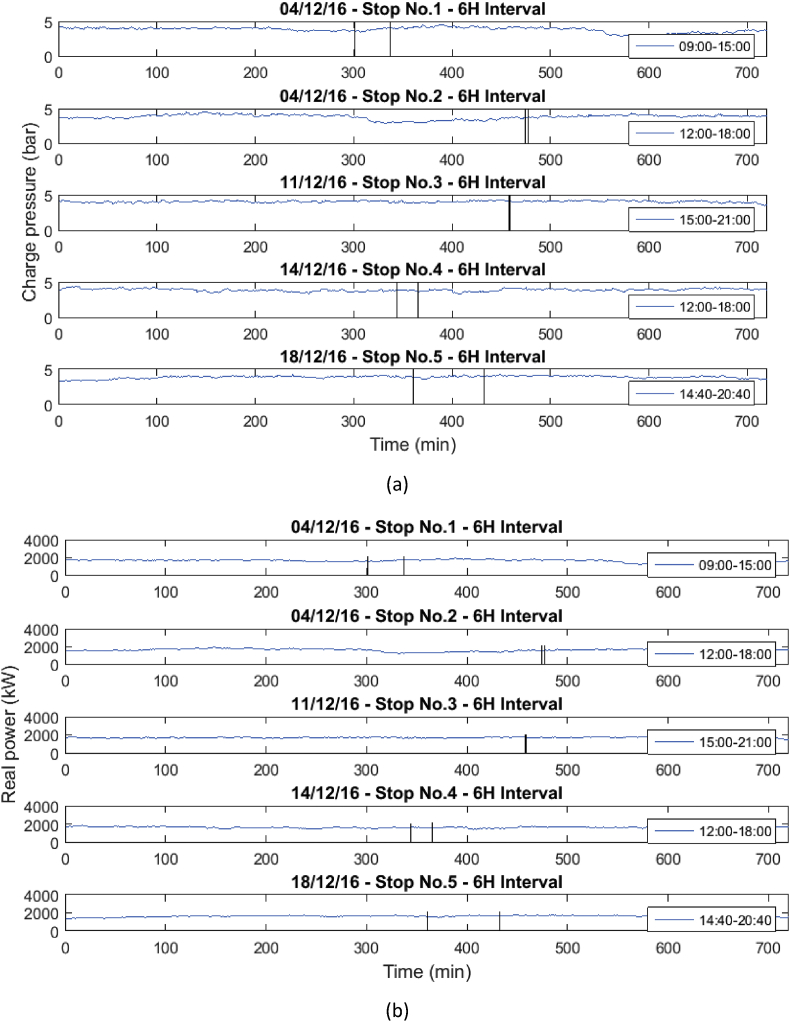
Behavior of the mean engine variables a) Charge pressure, and b) Real power.

**Fig. 4:**
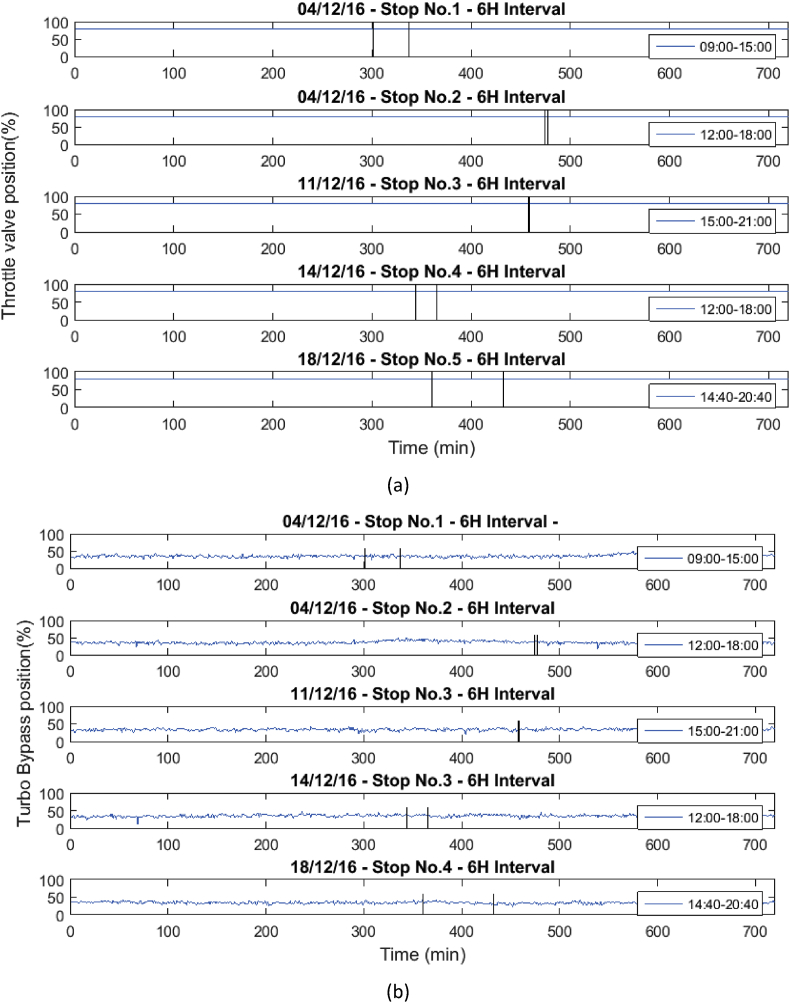
Behavior of the mean engine variables a) Throttle valve position, and b) Turbo bypass position.

**Fig. 5:**
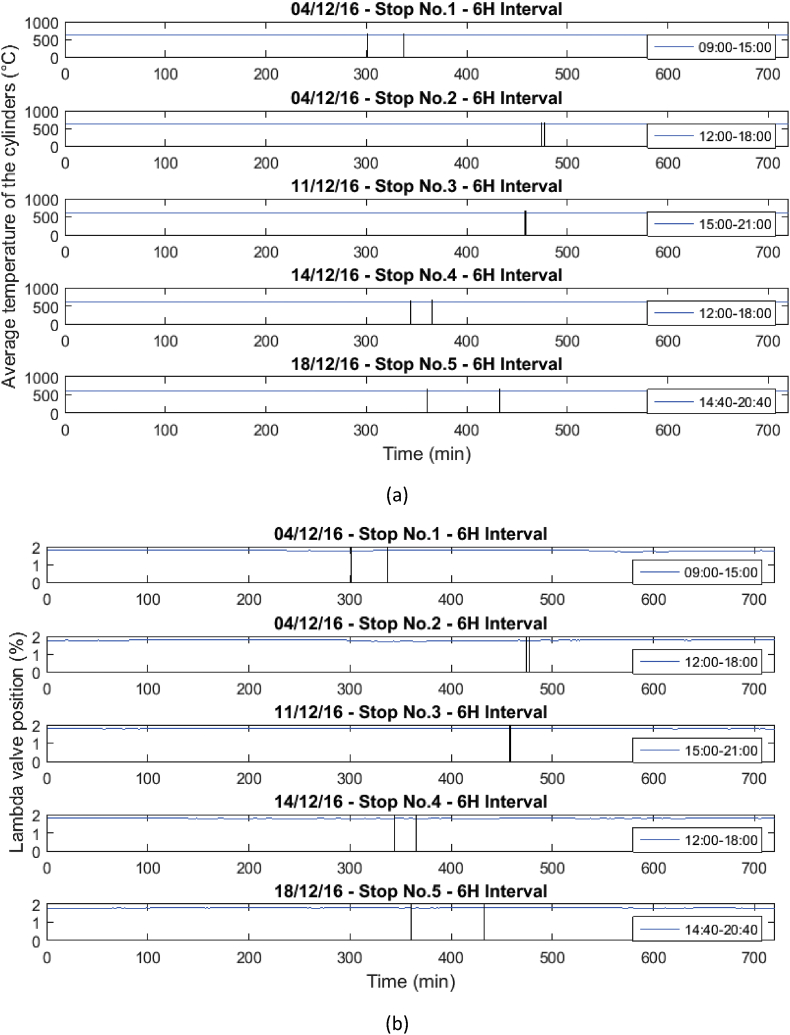
Behavior of the mean engine variables a) Average cylinder temperature, and b) Lambda valve position.

**Fig. 6:**
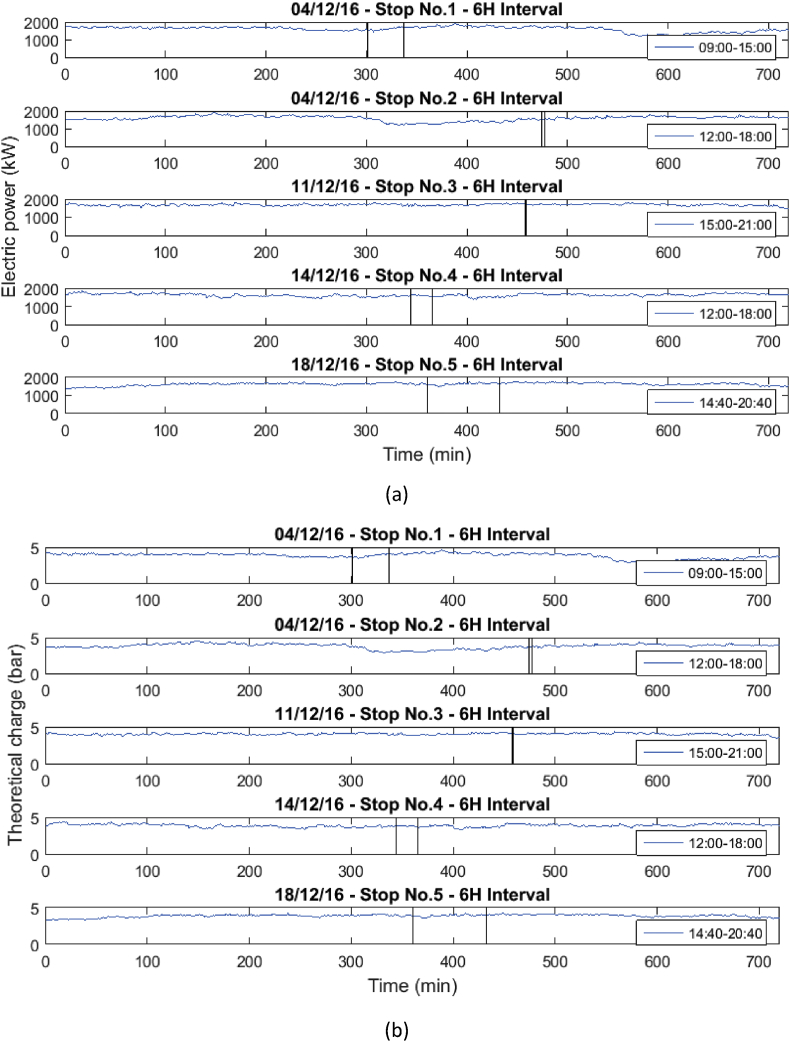
Behavior of the mean engine variables a) Electric power, and b) Theoretical charge.

**Fig. 7:**
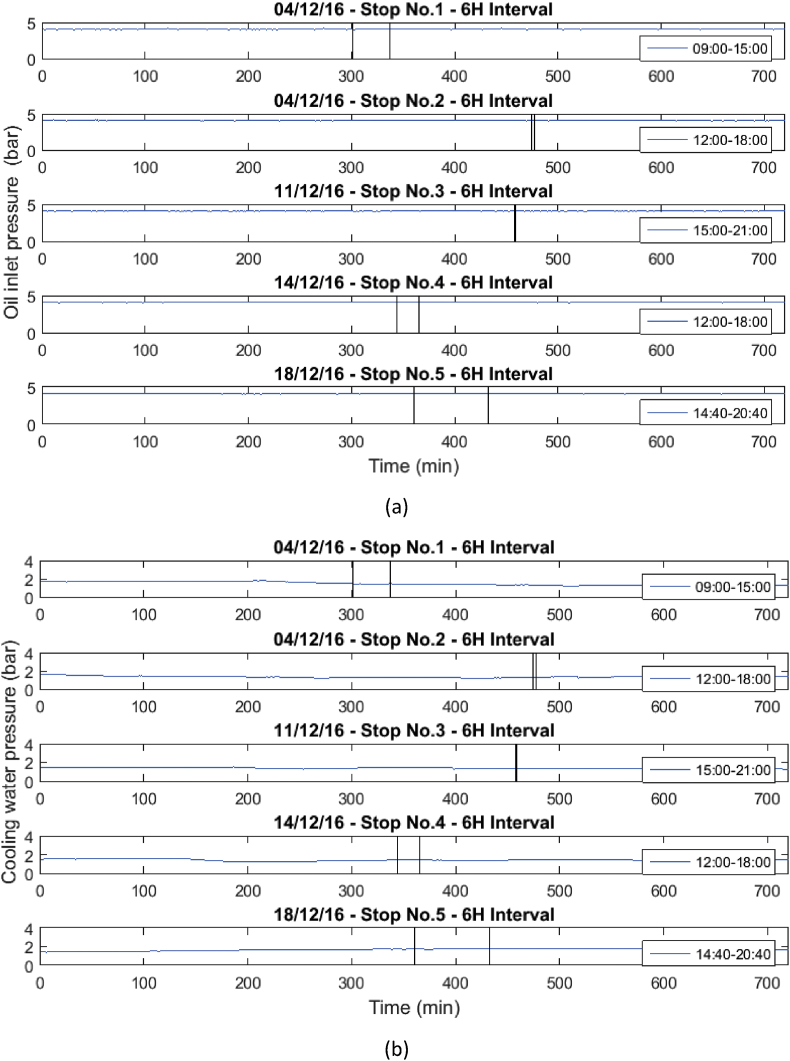
Behavior of the mean engine variables a) Oil inlet pressure, and b) Cooling water pressure.

**Fig. 8:**
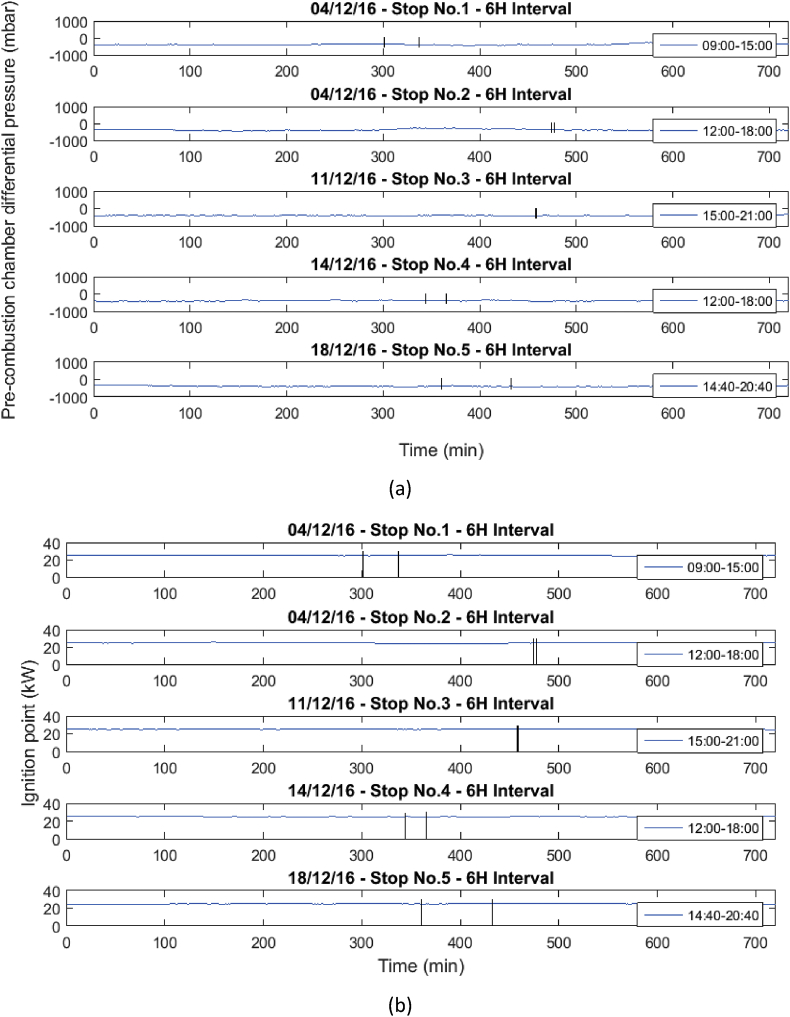
Behavior of the mean engine variables a) Pre-combustion chamber differential pressure, and b) Ignition point.

**Fig. 9:**
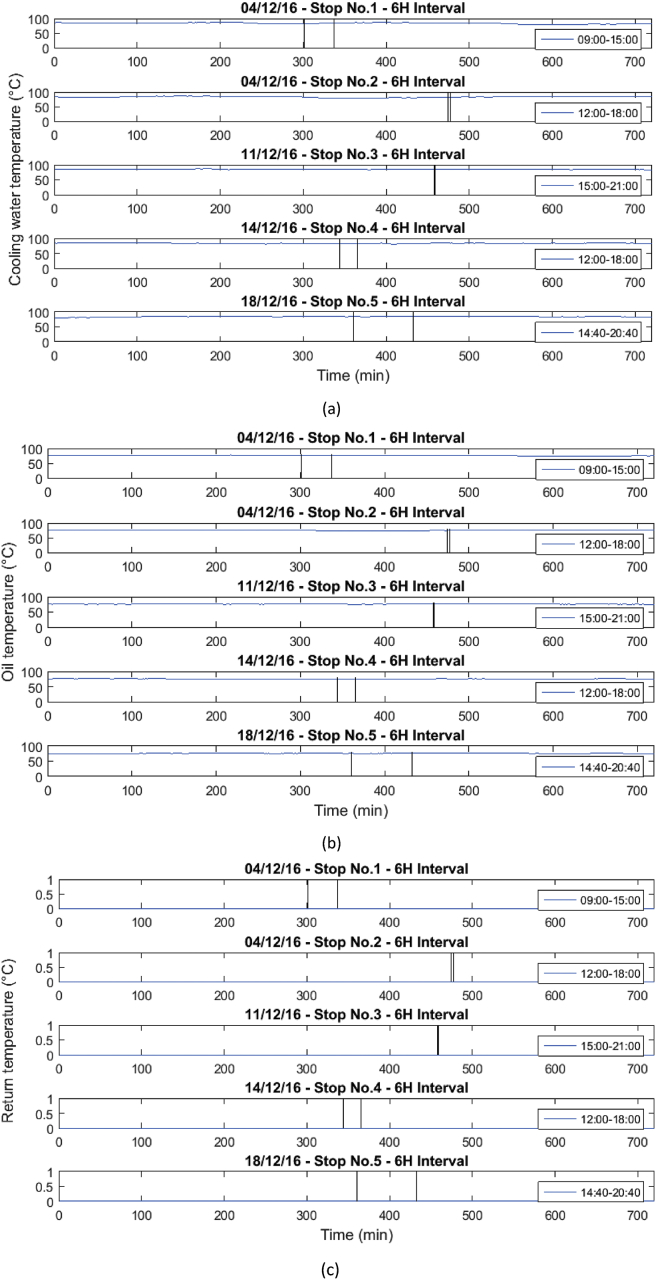
Behavior of the mean engine variables a) Cooling water temperature, b) Oil temperature, and c) Return temperature.

These operational data were taken when the gas engine was stopped due to failures in the power supply system or engine maintenance. Table 1 and Table 2 show the list of failures in the system, in which the operational data presented were taken. In Table 1, the stops presented in the engine will be presented, and in Table 2, the moments in which the engine stopped due to the disconnection of the external network system will be presented. Similarly, the behavior of the variables before, during and after the stops are presented in Fig. 1, Fig. 2, Fig. 3, Fig. 4, Fig. 5, Fig. 6, Fig. 7, Fig. 8, Fig. 9 as follow. The data raw to generate all these figures and tables were made with the data presented in Appendix A.

**Table 1:** Stops due to engine failure.

Generator output									
Number of stops	Failure code	Horometer	Date	Stop time	Start time	Time (hours)	Accumulated time	Affects availability	Description
1	1234	37,191	13/11/2016	7:36	8:34	0.96	0.96	yes	Scheduled maintenance by change of spark plugs
2	1234	37,615	1/12/2016	1:15	1:28	0.21	0.21	yes	Spark plug change, position #3
3	1234	37,620	1/12/2016	6:00	15:00	9	9.21	yes	Maintenance 38,000
4	1234	38,093	21/12/2016	8:00	16:29	8.29	17.5	yes	Oil and cylinder head change for compliance with working hours

**Table 2:** Stop due to external power failure.

Public network room							
Number of stops	Date	Stop time	Start time	Time (hours)	Accumulated time	Description	Mode
1	4/12/2016	11:30	11:48	0.3	0.3	Network failure 27.2 V	Island
2	4/12/2016	14:57	14:58	0.1	0.4	Network failure 26.2 V	Island
3	11/12/2016	18:49	18:49	0	0.4	Network failure 28.4 V	Island
4	14/12/2016	14:52	15:02	0.1	0.5	Network failure 27.4 V	Island
5	18/12/2016	17:43	18:19	0.6	1.1	Network failure 27.8 V	Island

## 2 Experimental design, materials, and methods

### 2.1 Experiment set up

The study's engine is located in the city of Barranquilla, capital of the Department of Atlántico, located on the north coast of Colombia. This engine is used for power generation, which feeds an organization that is dedicated to the transformation and conversion of flexible packaging. The energy supply is complemented by the external network. The energy supply works only with the energy generated by the engine (island), only with the electrical energy from the network or with the sum of the energy generated and the energy from the network (in synchrony). This engine was equipped with some sensors, through which the operational data presented in this work was taken, such sensors are shown in Table 3.

**Table 3:** Sensors technical data.

Reference	Measurement	Range	Precision
M.05-TI-001	Suction temperature sensor	−40 to 1200 °C	±2.5 °C
E.01.QI-001	Fuel gas inlet flow	0 to 200 Lt/min	±0.1 Lt/min
E.08-PI-003	Suction pressure	- 1 to 1.5 bar	0.5 (f.s.d.); ±0.15% (f.s.d.)
E.08-PI-002	Boost pressure	0 to 10 bar	0.5 (f.s.d.); ±0.15% (f.s.d.)
E.08-PI-004	Cooler outlet pressure	0 to 300 PSI	0.5 (f.s.d.); ±0.15% (f.s.d.)
PI E.08.001	Charge Pressure	0 to 10 bar	0.5 (f.s.d.); ±0.15% (f.s.d.)
E.08-TI-001	Mixture Temperature	−40 to 1000 °C	±1.5 °C
E.02.001	Exhaust gas temperature	−40 to 900 °C	±1.5 °C

**Fig. 10:**
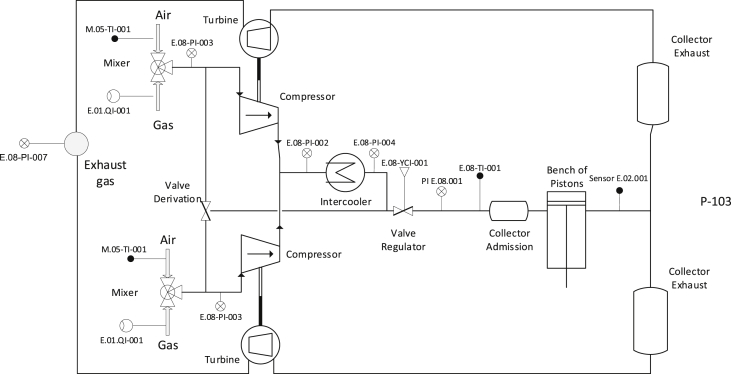
Schematic diagram of sensor mounting on the engine.

These measures were taken at the time of engine stops due to engine failure, or fluctuations in the state of the power supplied by the grid. These fault conditions were presented in Table 2, where the equipment's downtime is also reported. The schematic diagram of the sensors used to obtain the data presented in Appendix A, can be seen in Fig. 10.

### 2.2 Method

Under normal operating conditions, the combustible air mixture is generated at a line pressure of between 1.15 bar and 1.21 bar, and a volumetric flow rate of 110 L/s to 140 L/s, which are measured in the field and allow us to guarantee non-flammable operating lambda conditions. Thus, this value is regularly included in a normal operating condition between 1.4 and 1.8 [3].

The fuel then exits the mixer and enters the compressor stage of the turbochargers that run in parallel for the two engine intake lines. These admit the mixture by increasing both its pressure and temperature, to values between 3 bar and 5 bar, and temperature not measured in the process [4].

Next, the two flows are mixed to enter the engine intake manifold prior to cooling to lower its temperature, and then pass through the throttle valve, which allows the regulation of the mixture flow to the intake chamber according to its position, a point in the engine's process where the mixture enters the intake manifold and is distributed to the 12 cylinders, with a mixture temperature between 60 °C and 70 °C and load pressures between 2.6 bar and 4.6 bar [4].

In the engine control and safety system, a control system is programmed that generates warnings when the mixture temperature exceeds 75 °C, or the load pressures is higher than 4.6 bar. The data reported in this work corresponds to a condition before and after the failures [2].

The flow of the mixture can be regulated by the throttle valve and the turbo bypass valve. Thus, the throttle valve takes a percentage opening depending on the engine operation mode, which could be 80% for island mode, operation independent of the network, or 98% in synchronism, operation in parallel with the network. Therefore, the operational data where the electrical network is involved corresponds to an operation in motor synchronism. Additionally, the turbo bypass valve for its operation and control takes values between 15% and 50% independent of the operation mode. In this way, the flow supplied to the equipment and the power generated is regulated, which is between 1000 kW and 1979 kW [4].

**Fig. 11:**
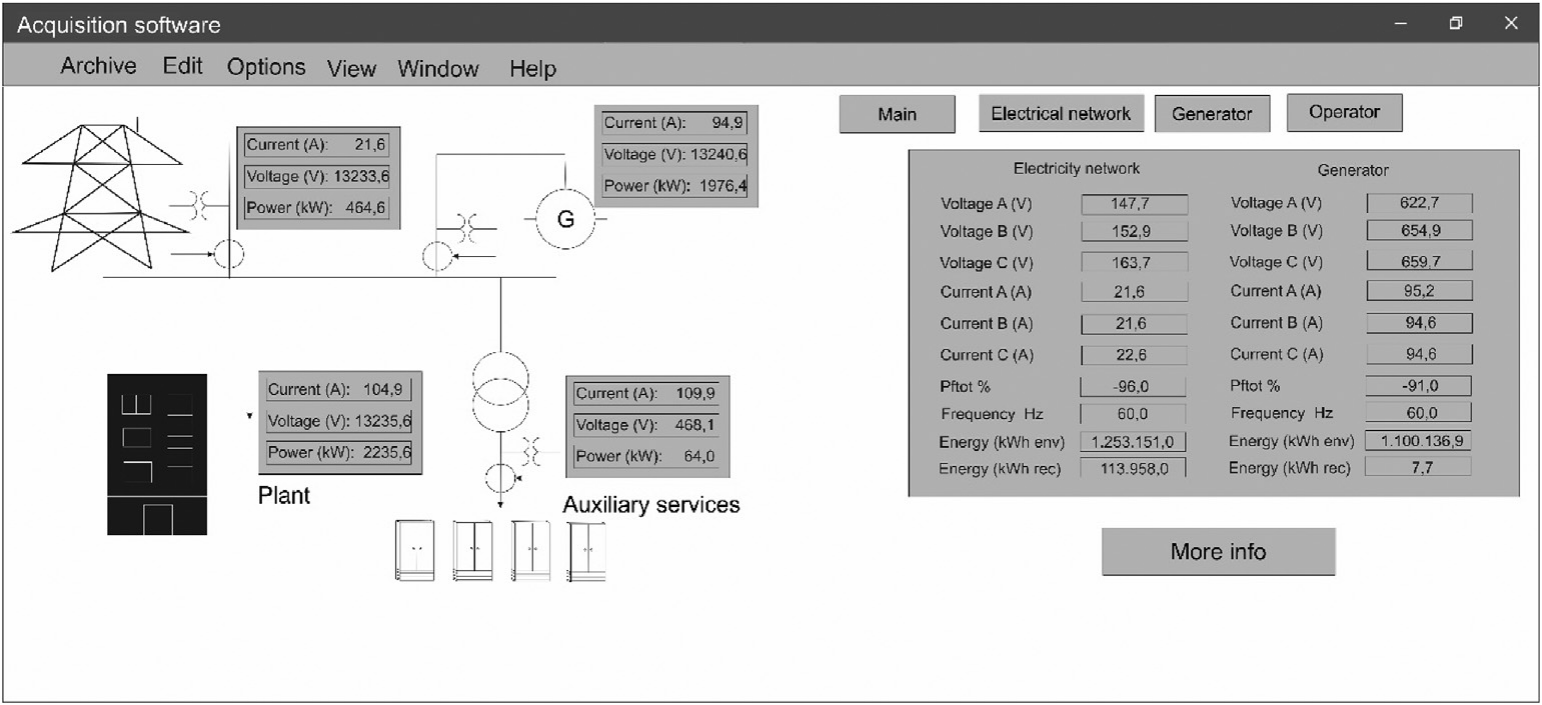
Main view data acquisition software.

Having knowledge of the normal behavior of the engine operation, it was decided to present the operational data taken before during and after failures presented in the engine or in the supply from the external electric network. These data were taken every 10 seconds, and they come mainly from the instrumentation of the generation engine, which corresponds mainly to different variables among which are: Load pressure, Boost pressure, Load temperature, Cylinder temperature, and Engine speed as shown in Fig. 11.

Data acquisition software was used to collect these data, in which electrical and mechanical data of the system that supplies the plant with energy are recorded. Fig. 11 shows the graphical user interface of the software used for data acquisition.
